# Circulating Biomarkers in Neuromuscular Disorders: What Is Known, What Is New

**DOI:** 10.3390/biom11081246

**Published:** 2021-08-20

**Authors:** Andrea Barp, Amanda Ferrero, Silvia Casagrande, Roberta Morini, Riccardo Zuccarino

**Affiliations:** 1 NeuroMuscular Omnicentre (NeMO) Trento, Villa Rosa Hospital, Via Spolverine 84, 38057 Pergine Valsugana, Italy; amanda.ferrero@centrocliniconemo.it (A.F.); silvia.casagrande@centrocliniconemo.it (S.C.); roberta.morini@centrocliniconemo.it (R.M.); riccardo.zuccarino@centrocliniconemo.it (R.Z.); 2 Department of Neurosciences, Drug and Child Health, University of Florence, Largo Brambilla 3, 50134 Florence, Italy

**Keywords:** neuromuscular disease, creatine kinase, antibody, neurofilament, microRNA, biomarker

## Abstract

The urgent need for new therapies for some devastating neuromuscular diseases (NMDs), such as Duchenne muscular dystrophy or amyotrophic lateral sclerosis, has led to an intense search for new potential biomarkers. Biomarkers can be classified based on their clinical value into different categories: diagnostic biomarkers confirm the presence of a specific disease, prognostic biomarkers provide information about disease course, and therapeutic biomarkers are designed to predict or measure treatment response. Circulating biomarkers, as opposed to instrumental/invasive ones (e.g., muscle MRI or nerve ultrasound, muscle or nerve biopsy), are generally easier to access and less “time-consuming”. In addition to well-known creatine kinase, other promising molecules seem to be candidate biomarkers to improve the diagnosis, prognosis and prediction of therapeutic response, such as antibodies, neurofilaments, and microRNAs. However, there are some criticalities that can complicate their application: variability during the day, stability, and reliable performance metrics (e.g., accuracy, precision and reproducibility) across laboratories. In the present review, we discuss the application of biochemical biomarkers (both validated and emerging) in the most common NMDs with a focus on their diagnostic, prognostic/predictive and therapeutic application, and finally, we address the critical issues in the introduction of new biomarkers.

## 1. Introduction

**Figure 1:**
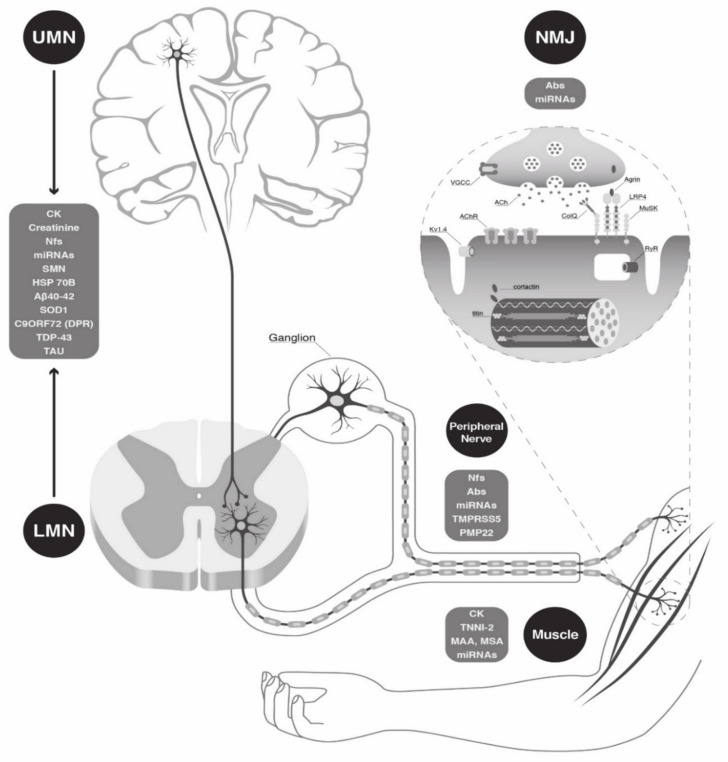
Main components of the neuromuscular unit and related biomarkers. Schematic representation of each part of the neuromuscular unit (upper motor neuron, lower motor neuron, neuromuscular junction and muscle) and of the main related circulating biomarkers. Abbreviations: Abs, antibodies; Aβ40-42, Amyloid-β40-42; AchR; CK, creatine kinase; DPR, dipeptide repeat; HSP70B, heat shock 70 kDa protein 7; LMN, lower motor neuron; NMJ, neuro muscular junction; MAA, myositis-associated Abs; miRNAs, microRNAs; MSA, myositis-specific Abs; PMP-22, peripheral myelin protein 22; Nf, neurofilament; SMN, survival motor neuron; SOD1, superoxide dismutase 1; TAR DNA-binding protein 43 (TDP-43); TMPRSS5, transmembrane protease serine 5; TTNI, serum troponin; UMN, upper motor neuron.

In the neuromuscular field, the urgent need for new therapies for some devastating diseases, such as Duchenne muscular dystrophy (DMD) or amyotrophic lateral sclerosis (ALS), has led to an intense search for new potential biomarkers. A biomarker should be quantifiable, reproducibly measurable with small coefficients of variation, and, whenever applied in the therapeutic field, it should predict the treatment response in a shorter timeframe than existing outcome measures. Evaluations of progressive disorder through measurable non-invasive biomarkers will provide clinicians with an invaluable tool for the care of patients affected by neuromuscular diseases (NMDs) [1]. In general, a biomarker, or biological marker, is a measurable indicator of some biological state or condition. Different types of biomarkers can be classified based on their clinical value into different categories: diagnostic biomarkers confirm the presence of a specific disease, prognostic biomarkers provide information about disease course, and therapeutic biomarkers are designed to predict (predictive biomarker) or measure treatment responses (surrogate biomarker) [2]. Therapeutic biomarkers, also called pharmacodynamic biomarkers, highlight responses to treatment and can be used to show whether a missing protein is restored after therapy. “NMDs” is a broad term including many diseases that can impair the functioning of a specific component of the neuromuscular unit: skeletal muscle (genetic myopathies, e.g., Duchenne muscular dystrophy/DMD, limb girdle muscular dystrophies/LGMDs, myotonic dystrophy type 1 and 2/DM1 and DM2, idiopathic inflammatory myopathies/IIMs), neuromuscular junction/NMJ (myasthenia gravis/MG; Lambert–Eaton myasthenic syndrome/LEMS), peripheral nerve (genetic neuropathies and dysimmune neuropathies, e.g., Charcot–Marie–Tooth neuropathies/CMTs, Guillain–Barré syndrome/GBS and chronic inflammatory demyelinating neuropathy/CIDP) or motor neuron disease (spinal muscular atrophy/SMA; amyotrophic lateral sclerosis/ALS) (Figure 1)**.** In the present review, we discuss the state of the art on the study of circulating biomarkers, with a focus on the main subclasses such as “easy-to-access” biomarkers in clinical practice (creatine kinase/CK, creatinine), autoantibodies (Abs), neurofilaments (Nfs) and microRNAs (miRNAs), emphasizing the role (diagnostic, prognostic/predictive, therapeutic) that each of them can play in the most frequent NMDs (starting from muscle, back to the motor neuron, through the NMJ and peripheral nerve) (Table 1).

## 2. Circulating Biomarkers

### 2.1. “Easy-to-Access” Biomarkers in Clinical Practice

#### 2.1.1. CK in Muscular Dystrophies and in IIMs

CK is an enzyme that catalyzes the conversion of creatine and triphospate to phospocreatine and viceversa [88].The cytosolic CK enzymes consist of two subunits, which can be either B (brain) or M (muscle) types. These two dimeric forms can exist in three different isoenzymes: CK-MM, CK-BB and CK-MB [89]. Both MM and MB increase in cardiac muscle involvement such as myocardial injury, where CK represents the main diagnostic biomarker before troponin T becomes available. Instead, in skeletal muscle injury or inflammation, total CK increases with the mostly MM form. 

In normal conditions, modest release into the blood stream is associated with moderate-intensity physical exercise. When a muscle damage occurs, a greater amount of CK is released in bloodstream [88]. CK is a common and frequently used blood test for patients presenting with muscular weakness, myalgia, or (in the case of young people) developmental delay [90]. 

In dystrophinopathies and many muscular dystrophies, CK represents a sensitive biomarker because elevated blood levels (up to 10- and 100-fold greater than the upper limit) indicate severe muscle damage [3]. CK is rather unspecific because plasma levels are also elevated in many forms of other muscular damage and levels are also influenced by other factors such as muscle mass, age, ethnicity, and muscle activity. Once elevated CK levels are found in plasma, genetic testing needs to be performed to confirm or exclude dystrophinopathies. The lack of tissue specificity, its day-to-day variability, and its decrease with age (reflective of progressive muscle loss) has limited its value as a therapeutic biomarker [91]. A possible way to overwhelm these limitations as a pharmacodynamic biomarker could be its application limited to the MM isoform level, just in early stages of disease (such as ambulant patients) through blood samples taken at the same time of the day (e.g., in the morning, just before taking steroids).

Serum CK is elevated in at least 90% of patients with dermatomyositis (DM) [14]. Its level can be normal even in individuals who are markedly weak, particularly in childhood DM, in patients with slow insidious disease, and in those with little residual muscle mass. The serum CK level is elevated fivefold or more in most polymyositis (PM) cases, in particular during the active phase, unlike DM and inclusion body myositis (IBM), in which CK can be normal [14]. In IIMs, serum CK can be used as a pharmacodynamic biomarker to monitor responses to therapy, but only in conjunction with physical examinations, because the CK level does not necessarily correlate with the degree of weakness, as observed in DM [20].

#### 2.1.2. CK in ALS

Serum CK represents a useful biomarker of muscle denervation, because it is increased in the plasma of patients affected by ALS and correlates with survival in some studies [81,92,93]. Conversely, some reports found no significant difference in survival in patients with a raised CK compared with those with normal levels [94,95]. This discrepancy may be attributed to differing rates of disease progression. Indeed, along with weight loss, alkaline phosphatase and albumin decrease, CK decline has been demonstrated to predict slow vs. fast disease progression [80].

#### 2.1.3. Creatinine in Motor Neuron Diseases

Creatinine is a breakdown product of creatine phosphate from muscle and protein metabolism. Serum creatinine is an important indicator of kidney health that is excreted unchanged by kidneys. In addition, creatinine can be considered a useful biomarker in some NMDs such as SMA. A recent study showed that serum creatinine seems to correlate with disease severity, SMN2 copy number, motor function, and denervation amount [61]. However, creatinine production strictly depends on muscle mass, liver function, diet and glomerular filtration [96]. Moreover, two recent studies found liver damage in a mouse model of severe SMA and kidney damage with renal tubular dysfunction in infants with type 1 SMA, reducing the reliability of using serum creatinine as an authentic SMA biomarker [97,98]. Plasma creatinine has also been reported to be a robust biomarker of disease progression in ALS, suggesting its potential role as a surrogate endpoint in clinical trials. In fact, it appeared to be a strong and independent predictor of mortality in ALS at 18 months, not influenced by the site of disease onset, and was comparable to the ALSFRS-R score [99]

### 2.2. Antibodies

Abs play an important role in NMDs with an inflammatory pathogenesis, such as IIMs (DM, PM and IBM), NMJ disorders (MG and LEMS) and dysimmune neuropathies (GBS and its variants, and CIDP). 

#### 2.2.1. Antibodies in IIMs

An increasing number of different Abs have been recognized in IIMs. They can be classified into two main categories: myositis-specific Abs (MSA) and myositis-associated Abs (MAA) [100]. MSA are highly specific for IIMs and enable the identification of specific IIM subtypes, representing a valid diagnostic biomarker for IIMs themselves and for IIM subgroups [16,17]. Different forms of IIMs have different systemic involvement and paraneoplastic risk; therefore, MSA can be also considered a prognostic biomarker. The most famous example of MSA are anti-synthetase Abs, associated with IIM, and with lung involvement in the so-called anti-synthetase syndrome. MAA, conversely, can also be detected in other connective tissue disorders regardless of muscular involvement, and are thus less useful as diagnostic biomarkers. However, they identify a peculiar clinical spectrum and may be considered prognostic biomarkers for systemic involvement in patients with already established myositis [17,18]. Several methods exist for MSA and MAA testing, with variable accuracy. The standard assay depends on the Abs subtype and may be different for clinical or research settings. The most widely used tests are usually commercial immunoenzymatic tests, such as an enzyme-linked immunosorbent assay (ELISA) [17]. Some Abs are considered alternately in MSA or in MAA group. An example is anti-cytosolic 5′-nucleotidase 1A Abs (anti-cN1A) in IBM [15]. The sensitivity of anti-cN1A in IBM significantly varies in different studies, ranging from 33% to 76%, mainly due to different detection methods and cut-off thresholds; specificity ranges from 87% to 100%, even though anti-cN1A Abs were also found in the sera of patients with other IIMs and other autoimmune diseases (mainly systemic lupus erythematosus and Sjögren syndrome) [101,102,103,104]. The presence of anti-cN1A Abs can represent a prognostic biomarker of risk of more severe dysphagia [19]

#### 2.2.2. Antibodies in NMJ Diseases

The most characterized biomarkers for MG are serum Abs targeting the post-synaptic end-plate of the NMJ [105]. Abs against acetylcholine receptor (AChR), muscle-specific kinase (MuSK) and lipoprotein-related protein 4 (LRP4) are the most well-established diagnostic biomarkers, but in the last decade, many new Abs with different clinical relevance have been discovered. 

AchR Abs are highly specific for MG, with a variable sensitivity depending on the MG subgroup: approximately 50% for ocular MG and 80–90% for generalized MG [106]. They are usually detected by a radioimmunoprecipitation assay that also enables quantification of their levels [107]. Sensitivity can be increased by using a cell-based assay (CBA), thus enabling the detection of Abs against clustered Ach and low-affinity Abs. CBA is not yet commercially available and standardized, but should be considered a second-level test given that it detects AChR Abs in up to 66% of considered seronegative MG [108]. AChR Abs are directly pathogenic because they may impair signal transduction through variable mechanisms of actions. They can activate a complementary cascade causing AChR loss, cross-link receptors leading to internalizations (antigenic modulation), and interfere with receptor activation by blocking Ach binding site [105,109]. AChR titers do not represent a prognostic or predictive biomarker because the Abs concentration does not correlate with disease severity [110]. A correlation between Abs titer fluctuation and clinical course in individual patients has been reported, and an increase in Abs concentration seems to predict exacerbations [22]. AChR concentration has therefore been used in different studies as a marker for treatment response. Despite this, the utility of AChR Abs concentration as a surrogate endpoint is still under debate [105,111]. MuSK Abs are found approximately in 40% of patients without AChR Abs [23]. They are tested using radioimmunoassay or ELISA, but CBA may be used to increase sensitivity in a research setting [105,112].They belong to the IgG4 subclass and inhibit agrin-induced AChR clustering without activating a complementary cascade [113]. MuSK and AChR abs rarely occur in the same patients. MuSK Abs identify a different MG entity with an atypical clinical picture, such as predominant bulbar weakness, relative sparing of ocular muscles, and lack of thymus alterations [24,25]. They are associated with a poor response to cholinesterase inhibitors and a good response to specific immunosuppressive therapy, such as rituximab [24,25,27]. A correlation between MuSK Abs concentration and disease severity/treatment response has also been demonstrated [26]. They can thus be considered not only diagnostic, but also prognostic and predictive biomarkers, with therapeutic usefulness in guiding treatment decisions. LRP4 Abs are detectable in around 19% of patients with double-negative MG, and they correlate with milder and often purely ocular symptoms [28]. They are usually detected by CBA, but no validated commercial test is yet available [28,107]. LRP4 Abs can also be found in MuSK- or AchR-positive MG with a more severe clinical picture [28]. Their roles as diagnostic and prognostic biomarkers are thus limited to double-negative MG. Their specificity for MG is low; they have also been reported in ALS patients without MG signs [114,115]. Agrin Abs have been detected by different assays (ELISA or CBA) in seronegative MG, along with AChR, MuSK or LRP4 Abs [116,117]. They can also be found in ALS patients [115]. Similarly, cortactin Abs have been detected by ELISA in a minority of patients with MG with or without AChR Abs, but they can also be found in myositis [118,119]. Finally, one study using a specific CBA reported the presence of collagen Q (ColQ) Abs in 3% of MG patients with or without other Abs, but they were also present in 2.3% of healthy subjects [120]. ColQ protein is concentrated in the extracellular matrix of the neuromuscular junction and anchors acetylcholinesterase (which breaks down Ach), but further studies are needed to clarify the pathogenetic role of these antibodies, which has not yet been demonstrated [120]. The low sensitivity and the lack of specificity of agrin, cortactin and ColQ ABs do not enable their use as diagnostic biomarkers. Anti-voltage-gated potassium channel Kv1.4 Abs have been reported in a Japanese cohort with a strong correlation with cardiac involvement [31]. This correlation was not confirmed in a Caucasian cohort [29]. Both studies used the same radioimmunoprecipitation assay. More data are thus needed to evaluate their role as a prognostic biomarker. Titin and ryanodine receptor (RYR) Abs are found in some patients with AChR Abs, mostly in association with thymoma and, less frequently, in non-paraneoplastic late-onset MG with severe disease [30,110]. Their role as a biomarker is thus restricted to patients younger than 50 years, where they strongly indicate the co-existence of a thymoma. Titin Abs are routinely detectable by ELISA, whereas no commercial kits are yet available for RYR, and only few diagnostic laboratories offer this testing for clinical practice [107].

The most well-known diagnostic biomarkers for LEMS are P/Q-type voltage-gated calcium channel (VGCC) Abs, which may be tested using a commercially available radioimmunoprecipitation assay kit. They are detectable in more than 90% of patient with a slight predominance in paraneoplastic forms [35,36]. They are highly sensitive but less specific, and a negative test cannot exclude the disease. They are also a specific diagnostic biomarker for paraneoplastic cerebellar degeneration, where they can be detected in up to 40% of cases, half of them with coexisting LEMS [121]. Raised levels of P/Q-type VGCC Abs at low titers have also been reported in patients with small cell lung cancer (SCLC) without neurological manifestations and, less frequently, in LEMS with different tumors [35]. Abs anti N-type VGCC can be detected by radioimmunoassays in 33–76% of paraneoplastic LEMS, always in association with P/Q-type VGCC. Their frequency is much lower in non-SCLC patients [35,36,38]. Another similar biomarker is SOX1 Abs. Such Abs have been detected in up to 64% of patients with SCLC and LEMS, and in no patients with non-paraneoplastic LEMS [37]. In patients with LEMS, the detection of SOX1 Abs or other onconeural (Hu and SOX2) Abs predicts the presence of SCLC with a sensitivity of 67% and a specificity of 95% [39]. Finally, GABA B receptor Abs are more frequently found in SCLC-associated than in non-paraneoplastic LEMS, particularly in the absence of limbic encephalitis [38]. All these Abs may be assessed using commercial kits for immunofluorescence/immunoblot and CBA for onconeural and GABA B receptor Abs [122,123]. They are thus considered important prognostic biomarkers for paraneoplastic LEMS and are useful to more closely follow those patients with no evidence of cancer at the time of diagnosis.

#### 2.2.3. Antibodies in Dysimmune Neuropathies

Gangliosides, which are composed of a ceramide attached to one or more sugars (hexoses) and contain sialic acid (N-acetylneuraminic acid) linked to an oligosaccharide core, are important components of the peripheral nerves. Four gangliosides (GM1, GD1a, GT1a, and GQ1b) Abs are associated with GBS and its variants [46]. IgG Abs to GM1 and GD1a are detectable in acute motor axonal neuropathy (AMAN) and its subtypes, acute motor-sensory axonal neuropathy (AMSAN) and acute motor conduction-block neuropathy, respectively, but not in the most frequent form of GBS in Caucasian populations, acute inflammatory demyelinating polyneuropathy (AIDP) [47]. IgM Abs against GM1 are instead associated with multifocal motor neuropathy with conduction blocks (MMNCB) [48]. Anti-GM1 Abs may be helpful at the onset of disease as a diagnostic biomarker to differentiate multifocal motor neuropathy from amyotrophic lateral sclerosis, but its sensitivity is low [46]. IgG Abs to GQ1b, which cross-reacts with GT1a, are strongly associated with Miller Fisher syndrome (MFS), its incomplete forms (acute ophthalmoparesis and acute ataxic neuropathy) [124]. Patients with pharyngeal–cervical–brachial weakness are more likely to have IgG anti-GT1a antibodies, which may cross-react with GQ1b; they are also less likely to have IgG anti-GD1a antibodies, which suggests a link to the axonal GBS [49]. The diagnostic utility of these Abs is often limited by their low sensitivity and by the lack of standardized assays. Thin-layer chromatography is the gold standard and reference method, but is unavailable for routine diagnostics. For this reason, commercial ELISA remains the most widely available and well-standardized assay [125].

Anti-myelin-associated glycoprotein (MAG) Abs are detectable by ELISA in half of IgM paraproteinemic neuropathy cases; they identify a pathognomonic clinical picture characterized by a sensory ataxic demyelinating neuropathy with neuropathic tremor and a slow progression [50]. Thus, they represent not only a diagnostic, but also a prognostic biomarker. However, previous studies have demonstrated no association between anti-MAG Abs titers and disease severity [126,127]. In addition, they can also be used as predictive and therapeutic biomarkers because the anti-MAG Abs titer correlates with the efficiency of rituximab response (anti-CD20) [51].

### 2.3. Neurofilaments 

Nf proteins belong to the type IV intermediate filament family, and they are components of the neuronal cytoskeleton [128]. They consist of three subunits: neurofilament light chain (NfL, 68 kDa), medium chain (NfM, 150 kDa) and heavy chain (NfH, 200 kDa). These subunits assemble into a complex of varying molecular mass. Nfs play an important role in several neuronal processes: cellular differentiation, control of axonal diameter of myelinated axons, axon outgrowth and regeneration [129,130]. Nfs (in particular NfL and NfH) are subjected to post-translational modifications, such as phosphorylation, that control their transport, function and degradation [131]. Nf levels have been used as biomarkers in many NMDs (e.g., dysimmune neuropathies and CMTs, SMA and ALS) to establish axonal damage and degeneration, and neuronal loss. They can be detected in blood because when axonal damage and neuronal death occur, Nfs are released and delivered into the cerebrospinal fluid (CSF), which are then circulated into blood [59,132,133]. Various assays have been developed for Nf testing in clinical or research settings such as ELISA, electrochemiluminescence (ECL) and Single Molecule Array (SIMOA) [134]. 

#### 2.3.1. Nfs in Dysimmune and Genetic Neuropathies

In a prospective study, baseline serum and CSF NfL levels of GBS patients were analyzed, with a focus on NfL levels and functional outcome at 1 year [53]. This study revealed higher NfL levels in GBS patients than in controls, both in serum and CSF. A correlation between serum NfL levels and Guillain–Barré Syndrome Disability Score and Inflammatory Rasch-built Overall Disability Scale (I-RODS) at every timepoint was observed. Patients with pure motor variant and Miller Fisher syndrome showed higher serum NfL levels than patients with sensorimotor GBS [53]. For this reason, serum NfL can be considered a promising prognostic biomarker in GBS. NfLs have also been demonstrated to be increased in CMT1A patients, where they are presumably released from degenerating axons [40].

#### 2.3.2. Nfs in Motor Neuron Diseases

In SMA 1 infants, raised plasma phosphorylated NfH (pNfH) levels have been identified compared to age-matched controls [59]; pNfH levels are inversely correlated with age and phenotypes severity. Furthermore, a marked reduction in pNfH levels in the serum of SMA 1 patients after nusinersen treatment has also been found. These data support pNfH levels as a promising biomarker for disease burden and response to nusinersen treatment [59,60]. pNfH levels decline with advancing age in both untreated patients and in healthy children; therefore, pNfH has a role as a biomarker in older patients [59]. Despite the significant differences found between SMA 1 and healthy controls, when considering milder clinical phenotypes (type 2 and 3), no significant differences seem to emerge in the levels of pNfH, in either child or adult patients compared to age-matched controls [135,136,137]. Furthermore, pNfH levels in CSF and plasma did not show any significant response to nusinersen treatment [135,136,137,138]. This is probably due to the depletion of motor neuron pools in the central nervous system which characterizes this chronic disease progression [138].

Raised Nf levels in the CSF and blood of ALS patients compared to healthy controls have already been demonstrated [139]. pNfH and NfL, in particular, play roles as diagnostic biomarkers in discriminating between ALS and ALS-mimics; pNfH has a higher sensitivity and specificity (90.7% and 88%, respectively) than NfL (sensitivity 85.4% and specificity 78.0%) [66,67]. However, the role of Nfs as diagnostic biomarkers for ALS when compared with diseases with elevated acute/subacute neuronal and axonal damage is limited [72]. In asymptomatic carriers of SOD1 and C9ORF72 mutations, Nfs increases both in CSF and in serum, prior to phenoconversion, confirm that increases in Nfs are already measurable early in the disease course of genetic ALS, and do not differ based on El Escorial diagnostic categories, highlighting the role of these biomarkers in early diagnosis [66,68,69,70,71]. There is a positive correlation between CSF NfL values and the number of regions affected by both UMN and LMN damage [66,140,141]. pNfH seems to correlate better with LMN loss and NfL with UMN loss, as confirmed by an imaging study which revealed that NfL levels in CSF correlate with the extent of corticospinal tract involvement on diffusion tensor imaging (DTI) [142]. In several studies, Nf levels in plasma and CSF showed a significant correlation with disease severity, such as a decline in the Amyotrophic Lateral Sclerosis Functional Rating Scale—Revised (ALSFRS-R), diagnostic latency, shorter survival, and time to generalization [69,73,74,75,76,143]. There is strong evidence that CSF NfL reflects overall disease aggressiveness in ALS, independent of disease accumulation, playing a prognostic role [144]. To date, no data exist concerning the fluctuation of Nf levels according to treatment response, but they are under evaluation as surrogate endpoints [66]. Whether the effect of riluzole on survival can be captured by measuring Nf levels remains unknown. 

### 2.4. microRNAs

Non-coding RNAs (ncRNAs) have emerged as relevant molecules in the pathogenesis of several human disorders. Among various ncRNAs, microRNAs (miRNAs) are secreted by many cell types and have already been used as biomarkers in several disease states [145]. miRNAs are small (~22 nucleotides) molecules that regulate gene expression at the post-transcriptional level [145]. miRNAs are easy to detect, stable in body fluids, and their expression levels reflect a distinct cell physiology state or damage to a specific tissue [146,147]. In addition, they are often contained in small vesicles (e.g., exosomes) that are released from cells and can traverse the blood–nerve barrier and be released into the general circulation [145].

A subset of muscle-enriched miRNAs, called myomiRs (miR-1, miR-133a, miR-133b, and miR-206), have been well described and investigated in NMDs. miR-1 and miR-133a are expressed from the same transcript within the skeletal and cardiac muscle, but they have different functions [148]. miR-1 promotes myogenesis and terminal differentiation, acting on histone deacetylase 4 (HDAC4) and connexin-43, whereas miR-133 enhances myoblast proliferation [149]. miR-206 and miR-133b are also codified by the same ncRNA. miR-206 is specific for skeletal muscle, and is particularly represented in oxidative fibers where it is expressed in proliferating myoblasts under the negative regulation of TGF-β and myostatin [150]. Moreover, miR-206 has a role in muscle hypertrophy and atrophy and suppresses utrophin, whereas its overexpression caused upregulation of utrophin levels in dystrophic conditions and decreased proinflammatory cytokines and macrophagic infiltration in mdx mouse muscle [151,152,153,154]. There are several techniques to detect miRNAs: microarray, quantitative real-time polymerase chain reaction (qRT-PCR)-based array, quantitative nCounter or next-generation sequencing (NGS), with the subsequent validation of identified miRNAs by qRT-PCR [155].

#### 2.4.1. microRNAs in Muscular Dystrophies 

Previous studies have demonstrated that myomiRs are increased in the serum of DMD patients and animal models of DMD (mdx mice) [5,6,156,157,158,159]. Although a correlation between myomiRs and disease progression in DMD is still unclear, an inverse correlation between myomiR levels in ambulant DMD patients and disease severity, evaluated through the Medical Research Council (MRC) scale, temporized tests or North Star Ambulatory Assessment (NSAA) scale, has been shown [6,157]. Opposite results emerged from another study, where lower myomiR levels in non-ambulant patients were detected compared to ambulant subjects [5]. In addition, the same authors found that DMD patients on a daily steroid regimen had higher levels of miR-1, miR-31 and miR-133b compared to patients on an intermittent regimen or absence of steroid treatment [5]. One possible explanation is that corticosteroids tend to increase muscle mass, suggesting a correlation between this anabolic effect and circulating miRNAs. Modifications in miRNA levels in the muscle or serum of DMD patients could represent a promising and non-invasive tool to evaluate the response of novel treatments which influence dystrophin expression, muscle damage and inflammation. mdx murine models treated with the skipping of exon 23 (through adeno-associated virus vectors) showed a complete normalization of increased miR-1 and miR-206 serum levels after 1 month from the administration; this result was also proportional to dystrophin restoration[6]. In the dystrophin restoration of mdx mice after morpholino-mediated treatment, a normalization of serum myomiRs was detected as well, and it was proportional to the degree of protein rescue [7]. Interestingly, exon 45 skipping in DMD myoblasts, which ensures the correct localization of nitric oxide synthase (NOS), has determines normalized miR-1 and miR-29c expression [8]. Therefore, myomiRs seem to represent a promising surrogate biomarker, because their levels can be normalized after dystrophin restoration in models of DMD.

miR-206 was also found to be significantly elevated in an LGMD patient cohort in comparison with a control group [10]. An over-expression of the same miRNA (50–80-fold) was detected in two patients with a severe and early disease course in transportinopathy (LGMD1F) and calpainopathy (LGMD2A). The functional impairment was observed clinically, and muscle loss and atrophy, documented by muscle MRI, provided the first evidence that miR-206 is associated with phenotypic expression and it could be used as a prognostic biomarker of LGMD disease progression [10].

myomiRs are also increased in DM1 patients compared with healthy subjects [11]. A downregulation to normal values of myomiRNAs after physical rehabilitation in DM1 patients was detected [13]. Seven of the miRNAs identified in DM1 patients (miR-1, miR-133a, miR-133b, miR-206, miR-140, miR-454, and miR-574) were also found to be downregulated in the plasma of a small group of DM2 patients [12].

#### 2.4.2. microRNAs in NMJ Diseases

The role of miRNAs as biomarkers in MG is still under evaluation, and further studies will be necessary. One recent study analyzing the serum circulating miRNAs profile of patients with MG and AChR Abs found a dysregulation of three different miRNAs: an increased level of miR-150-5p, involved in T cell differentiation, and of miR-21-5p, a regulator of Th1 versus Th2 cell response, and reduced levels of miR-27-3p, a downregulator of natural killer cell cytotoxicity[32]. They further evaluated the effect of thymectomy on miRNA expression, providing evidence of reduced levels of miR-150-5p in those patients with significant improvements in the MG clinical picture after surgery [32]. This correlation was later confirmed in another perspective study which found no change in miR-150-5p concentration in patients with steroid therapy only, suggesting a specific correlation with thymoma [160]. Nevertheless, another longitudinal study demonstrated that circulating miR-150-5p and miR-21-5p levels were lower in MG patients with immunosuppressive medications compared with naïve patients, regardless of disease severity [34]. The potential role of miRNAs as prognostic biomarkers has been showed in ocular MG, where higher levels of miR-30-5p predict progression to generalize MG [33]. A different profile of serum circulating miRNAs has been detected in MuSK MG patients, with increased let-7 family circulating miRNAs [161].

#### 2.4.3. microRNAs in Genetic Neuropathies

A recent study by Wang and colleagues confirmed elevated levels of myomiRs along with a set of other miRNAs that are highly expressed in the Schwann cells of peripheral nerves, in patients affected by CMT1A. Furthermore, the elevation of several of these miRNAs (e.g., miR-133a, miR-206, miR-223) could be used to discriminate cases from controls. They also found that NfL levels were most highly correlated with miR133a, and putative Schwann cell miRs (e.g., miR223, -199a, -328, -409, and -431) [45].

#### 2.4.4. microRNAs in Motor Neuron Diseases

Increasingly, studies suggest that miRNAs might act as essential modulators of SMN-mediated molecular pathways [149,162]. Abnormal expression levels of miR-9 and miR-206 in spinal cord, skeletal muscle, and sera from transgenic mice, and in sera from SMA patients, was observed [62]. These miRNAs were altered prior to neuroanatomical changes in spinal cord and skeletal muscle. In addition, a reduction in myomiRs under nusinersen treatment in pediatric SMA type 2 and 3 patients was detected, suggesting that myomiRs could potentially serve as informative biomarkers to monitor disease progression, and responses to antisense oligonucleotides (ASOs) therapy [63].

miRNAs are among the main epigenetic mechanisms involved in ALS disease development [163,164]. The principal miRNAs as promising ALS biomarkers are miR-206, miR-133b, miR-27a, miR-338p, miR-183, miR-451, let-7, miR-125: these have been reported to be commonly deregulated in multiple studies, but none of them are specific for ALS [85]. A combination of three pairs of miRNAs (miR-206/miR-338-3p, miR-9/miR-129-3p, and mi-R335-5p/miR-338-3p) were able to clearly distinguish between ALS and healthy subjects with a sensitivity of 84% and a specificity of 82%, with higher accuracy than could be achieved by an individual miRNA [86]. miRNAs could be used to identify “potential ALS cases” too. In fact, 22 of 30 downregulated miRNAs in ALS were also downregulated in presymptomatic genetic forms of ALS, with a greater downregulation after disease onset [165]. A recent longitudinal study showed that miR-206, miR-133a, miR-151a-5p, miR-199a-5p and miR-423-3p level fluctuations during disease progression can be considered good biomarkers to follow ALS course. miR-206 and miR-133a seem to have, together with miR-151a-5p, a good prognostic value, whereas miR-199a-5p and miR-423-3p are highly expressed, and consequently, are easily detectable [87]. In ALS patients, there is a weak correlation in miRNA expression between CSF and serum, probably due to the different mechanisms regulating miRNA levels in these two body compartments [165].

### 2.5. Gene Products

Downstream protein readouts linked to genetic mutations have been explored as biomarkers; particularly, the measurement of gene products is an attractive candidate for pharmacodynamic biomarkers.

#### 2.5.1. Gene Products in Genetic Neuropathies

Peripheral myelin protein 22 (PMP22) is a protein of 22 kDa (MIM #601097) expressed in myelin sheets of peripheral nerves. Increased expression of PMP22 represents the most likely molecular mechanism underlying CMT1A [42]. Reducing the production of PMP22 is considered the best way to positively impact disease progression [166]. PMP22 mRNA and protein levels are extremely variable among patients. [167,168,169]. For this reason, it appeared to not be a reliable tool in most studies to discriminate patients with CMT1A from healthy subjects, or as a surrogate endpoint A novel skin biopsy to precisely assess the PMP22 expression level (by RT-PCR and immunoelectron microscopy) as a candidate biomarker for CMT1A clinical trials has recently been developed [43,44]. 

#### 2.5.2. Gene Products in Motor Neuron Diseases

In SMA, clear differences in SMN levels in peripheral blood between SMA patients and controls which have been shown in type 1, type 2 and 3 and transcript levels are related to clinical severity [54,55,56]. Blood changes reflect variations observed in target tissues, suggesting that real-time data from peripheral blood can be used as a biomarker of disease progression, even if not all studies have demonstrated a statistical correlation between motor function and SMN levels [170,171,172,173,174,175,176]. Current therapeutic strategies against SMA aim to increase the amount of SMN produced by the SMN2 gene. Quantification of SMN mRNA or protein levels is the most applied molecular biomarker in monitoring therapeutic response [57,58]. RT-PCR assays and cell immunoassays can be used to measure the SMN levels in patient samples [56].

Superoxide dismutase 1 (SOD1) SOD1 levels are reported to be increased in the leukocytes of ALS patients [177]. They are usually detected by ELISA or Western blot [84]. Although most studies have only considered SOD1 levels as primary outcomes in CSF, SOD1 has been demonstrated to also be reduced in leukocytes in response to pyrimethamine treatment in genetic ALS with SOD1 mutation [77,83,84]. The hexanucleotide repeat expansion associated with C9ORF72 disease causes an accumulation of specific proteins called C9RAN dipeptide repeats (DPRs). Toxicity is thought to be in part due to the sequestration of RNA binding proteins [178]. Similarly to misfolded SOD1 protein, these DPRs are measurable in CSF [179]. A cross-sectional study showed that one of these, poly (GP), is detectable in the CSF of ALS patients with fronto-temporal-dementia (FTD) and C9ORF72 mutation but not in controls, and that levels are increased in pre-clinical stages [77]. This concept was further explored longitudinally, showing that DPR levels are stable over time, supporting their use as a pharmacodynamic biomarker [78]. This latter study also demonstrated that poly (GP) levels are reduced by using ASOs in C9ORF72 cell and mouse models [78]. These results suggest that RNA repeat mitigation could be a target in this disease subtype. Indeed, a clinical trial using anti-sense oligonucleotides to lower DPRs in human ALS patients with C9ORF72 mutations has been planned.

Neuronal and glial inclusions of TAR DNA-binding protein 43 (TDP-43) have been implicated in the pathogenesis of sporadic ALS and the linked FTD [180]. Subsequent studies have found elevated TDP-43 levels (by immunoblotting and quantitative mass spectrometry) in the CSF of ALS patients compared to healthy subject and patients with other neurodegenerative or neuroinflammatory diseases, and higher levels in ALS than in FTD [181,182,183]. However, its diagnostic accuracy has not yet been demonstrated. It has been reported that serum concentrations of TDP-43 are 200-fold higher than in CSF, suggesting the pharmacodynamic utility of serum TDP-43 diagnostics as a biomarker [183]. There is little available evidence for use as a marker of disease progression or prognosis, and longitudinal studies are needed.

### 2.6. Other Biomarker Proposals

Two different isoforms of serum troponin I (TNNI) exist: TNNI-1 and TNNI-2, expressed in slow and fast skeletal muscle fibers, respectively. In DMD patients, the pathological process (degeneration and regeneration changes) primarily affects the fast skeletal muscle fibers. A recent study analyzed the TNNI-2 isoform in a large cohort of subjects with DMD [4]. These authors reported increased levels of serum CK and TNNI-2 in healthy controls compared to DMD patients, supporting the hypothesis of early and selective fast skeletal muscle fiber involvement in dystrophinopathies. Conversely, serum TNNI-1 isoform levels were similar among DMD, Becker muscular dystrophy (BMD) patients and healthy controls, suggesting a relative sparing of slow skeletal muscle fibers [4]. These findings support the potential use of serum TNNI-2 as a therapeutic biomarker which could be used to detect treatment response in DMD patients receiving disease-modifying therapy.

IL-8 is a proinflammatory chemokine primarily secreted by circulating monocytes and local macrophages, with an essential role in the inflammatory process of inducing a chemotaxis gradient, migration, and oxidative burst [184]. CSF IL-8 concentration, when measured at the time of the initial diagnostic workup, has reportedly been significantly increased in GBS when compared with CIDP, suggesting that a measurement of IL-8 with a cutoff of >70 pg/mL yields a positive predictive value of 96% to differentiate patients with GBS from those with CIDP [52].

To identify additional plasma biomarkers for CMT1A, a screen of 460 unique proteins identified only NfL and a novel Schwann cell-derived protein, transmembrane protease serine 5 (TMPRSS5), to be consistently elevated in independent cohorts of CMT1A samples [41]. TMPRSS5 does not correlate to disease score, and because it is highly expressed in Schwann cells, its elevation may reflect the ongoing disease process. 

One of the new interesting proposals about SMA biomarkers comes from RNA sequencing and differential expression analyses performed in samples from SMA type 1 subjects <12 months old and age-matched healthy subjects [64]. For the first time, this analysis identified the heat shock 70 kDa protein 7 (HSP70B) as a potentially new biomarker to track SMA progression in the first year of life, indicating that its circulating protein levels are associated with NF levels in SMA newborns and infants.

Biomarkers of neuroaxonal degeneration have been investigate as potential biomarkers in SMA and ALS. Amyloid-β40 (Aβ40) and amyloid-β42 (Aβ42) peptides showed an increased level after nusinersen treatment in an adult cohort of patients with SMA types 2 and 3 [65]. These molecules have been already investigated as biomarkers in ALS [185]. Aβ-42 was increased in a large cohort of ALS patients in relation to control subjects, with a positive correlation between Aβ-42 levels and ALSFRS-R score [82].

Another emerging biomarker in ALS is the TAU protein, a molecule involved in the stabilization of neuronal microtubules. Phosphorylated tangles, with TAU as the major constituent, are seen in Alzheimer’s disease and ALS when associated with TDP-FTD. Raised total-TAU levels were reported in the CSF of ALS patients in one study, but no difference was found in another, and there was failure to replicate this quantification in a multi-center analysis [79,186,187].

## 3. Discussion

In the past, research interest in NMDs has mainly focused on diagnostic and prognostic biomarkers. More recently, with the advent of new therapies (e.g., ataluren and antisense oligonucleotides for DMD, nusinersen and risdiplam for SMA), extensive research efforts have been made to identify diagnostic biomarkers for preclinical diagnosis and potential therapeutic biomarkers. Disease biomarkers enabling diagnosis at preclinical stages could be interesting for the wide screening of potentially treatable NMDs (e.g., newborn screening for SMA). Predictive biomarkers are crucial to enable identification of those patients who are most likely to benefit from a treatment, and consequently, to determine patients’ eligibility for specific therapies. However, in this “gene therapy era”, the greatest effort should be made in the identification of a surrogate endpoint, essential for the correct conduction and safety assessment of most clinical trials. Circulating biomarkers, as opposed to instrumental/invasive ones (e.g., muscle MRI or nerve ultrasound, muscle or nerve biopsy), are generally easier to access, less “time-consuming”, and less operator-dependent; ongoing studies are thus focusing on this biomarker categories. For example, the detection of myomiR levels in the serum of DMD patients would certainly be easier to assess compared to the detection of dystrophin restoration checked on muscle specimens of patients treated with disease-modifying therapies. Again, the quantification of Nfs or myomiR levels in SMA or dystrophic patients, respectively, could be an easier surrogate biomarker to assess, when compared to imaging studies such as muscle magnetic resonance which can be difficult to perform, especially in ventilated patients or patients with metal bars who have undergone spinal surgery. In ALS patients, serial analysis of serum Nf levels might represent a valid alternative to electrophysiological studies such as Motor Unit Number Estimation (MUNE), which represents a demanding technique, often requiring good operator expertise.

However, most of the previously described therapeutic biomarkers do not completely satisfy these requirements. An example is the laboratory quantification of Nfs and miRNAs, which could be expensive, are often time-consuming, and are not always commercially available or standardized. Thus far, most validated biomarkers belong to the diagnostic and prognostic categories, and therapeutic biomarkers can be considered as still under evaluation. 

The search for new potential circulating biomarkers in fact often represents a difficult path to be pursued. Several fundamental points need to be considered: (i) specimen type (urine, blood or CSF); (ii) day-by-day variability, which can affect the time and the number of serial samples required to reliably track changes in biomarkers; (iii) stability, because biomarkers may require expensive and logistical cautions to guarantee temperature stability; (iv) reliable performance metrics (e.g., accuracy, precision and reproducibility), across laboratories; and finally (v) the clinical interpretation of results [188,189,190,191,192,193]. 

## 4. Conclusions

A balance between costs and benefits in the search of a specific biomarker must always be taken into careful consideration, in order to avoid an excessively extensive “biomolecule quantification” that could not add any new information regarding diagnosis, prognosis or response to therapy in clinical practice. Further research, especially longitudinal studies, are thus needed to unravel the real potentials of these biomarkers.

## Figures and Tables

**Table 1:** Validated (in black) and emerging (**in green**) biomarkers in the most common neuromuscular disorders classified by clinical application.

NMD			Therapeutic Biomarkers		Ref.
	Diagnostic Biomarkers	Prognostic Biomarkers	Predictive Biomarkers	Surrogate Endpoint	
**DMD**	CK **TTNI-2 **			**miR-1, -31, 29c, -133, -206 TTNI-2 **	[3,4,5,6,7,8]
**LGMDs**	CK	**miR-206 **			[9,10]
**DM1**	**miR-1, -133a, -133b, -206, -140, -454, -574 **			**miR-1,-133a, -133b, -206 **	[11,12,13]
**IIMs**	CK MSA (anti-cN1A) MAA	MSA (anti-cN1A) MAA		CK	[14,15,16,17,18,19,20]
**MG**	AChR Abs MuSK Abs LRP4 Abs **miR150-5p, 21-5p, 27a3p **	MuSK Abs Titin and RyR Abs **LRP4 Abs** **Kv1.4 Abs** **miR150-5p, -30e-5p**	MuSK Abs	AChR Abs titer MuSK Abs titer **miR-150-5p, 21-5p**	[21,22,23,24,25,26,27,28,29,30,31,32,33,34]
**LEMS**	P/Q-type VGCC Abs	SOX1 Abs N-type VGCC Abs Onconeural Abs **GABAB receptor Abs **			[35,36,37,38,39]
**CMT**	**NfL** **TMPRSS5** **PMP22** **miR133a, -206, -223 **			**TMPRSS5** **PMP22 **	[40,41,42,43,44,45]
**Dysimmune** **Neuropathies**	Gangliosides Abs MAG Abs **IL-8 **	MAG Abs **Gangliosides Abs** **NfL**	**MAG Abs **	**MAG Abs **	[46,47,48,49,50,51,52,53]
**SMA**	**SMN** **pNfH** **miR9, -206**	SMN Creatinine		**SMN** **pNfH ** **HSP70B** **Aβ40-42** **miR-133a, -133b, -206 and -1 **	[54,55,56,57,58,59,60,61,62,63,64,65]
**ALS**	**NfL, pNfH** **C9ORF72 (DPR)** **TDP-43** **TAU** **miR-206, -133-b, -27a, -338-p, -183, -451, let-7, -125, -9, -129-3p, -335-5p, -199a-5p, -423-3p **	CK Creatinine NfL, pNfH		**Creatinine** **Aβ-42** **SOD1** **C9ORF72 (DPR)** **miR-206, -133a, -151a-5p **	[66,67,68,69,70,71,72,73,74,75,76,77,78,79,80,81,82,83,84,85,86,87]

Abbreviations: Abs, antibodies; Aβ40, amyloid-β40; AchR, acetylcholine receptor; ALS, amyotrophic lateral sclerosis; CK, creatine kinase; cN1A, cytosolic 5′-nucleotidase 1A; DM1, myotonic dystrophy type 1; DMD, Duchenne muscular dystrophy; DPR, dipeptide repeat; HSP70B, heat shock 70 kDa protein 7; IIMs, inflammatory myopathies; IL-8, interleukine-8; LEMS, Lambert–Eaton myasthenic syndrome; LGMDs, limb–girdle muscular dystrophies; LRP4, lipoprotein-related protein 4; MG, myasthenia gravis; MAA, myositis-associated Abs; miRNA, microRNA; MSA, myositis-specific Abs; MuSK, muscle-specific kinase; NMD, neuromuscular disease; PMP-22, peripheral myelin protein 22; RYR, ryanodine receptor; SMA, spinal muscular atrophy; Nf, neurofilament; SMN, survival motor neuron; SOD1, superoxide dismutase 1; TAR DNA-binding protein 43 (TDP-43); TMPRSS, transmembrane protease serine 5; TTNI, serum troponin; VGCC, voltage-gated calcium channel.

## Data Availability

Not applicable.

## Abbreviations

AAV, adeno-associated virus; Abs, antibodies; Aβ40, amyloid-β40; Aβ42, amyloid-β42; AchR, acetylcholine receptor; AIDP, acute inflammatory demyelinating polyneuropathy; ALS, amyotrophic lateral sclerosis; ALSFRS-R, Amyotrophic Lateral Sclerosis Functional Rating Scale—Revised; AMAN, acute motor axonal neuropathy; AMSAN, acute motor-sensory axonal neuropathy; anti-cN1A, anti-cytosolic 5′-nucleotidase 1A Abs; ASO, antisense oligonucleotides; B, brain; CBA, cell-based assay; CIDP, chronic inflammatory demyelinating neuropathy; CMTs, Charcot–Marie–Tooth neuropathies; CK, creatine kinase; cN1A, cytosolic 5′-nucleotidase 1A; ColQ, anti-collagen Q; CSF, cerebrospinal fluid; DM, dermatomyositis; DM1, myotonic dystrophy type 1; DM2, myotonic dystrophy type 2; DMD, Duchenne muscular dystrophy; DPR, dipeptide repeat; DTI, diffusion tensor imaging; ELISA, enzyme-linked immunosorbent assay; FTD, fronto-temporal-dementia; GABA, gamma-aminobutyric acid; GBS, Guillain–Barré syndrome; GD1a, ganglioside disialic; GM1, ganglioside monosialico; GQ1b, ganglioside quatersialico; GT1a, ganglioside trisialic; HDAC4, histone deacetylase 4; HSP70B, heat shock 70 kDa protein 7; IBM, inclusion body myositis; IIMs, inflammatory myopathies; IL-8, interleukine-8; I-RODS, Inflammatory Rasch-built Overall Disability Scale; LEMS, Lambert–Eaton myasthenic syndrome; LGMDs, limb–girdle muscular dystrophies; LMN, lower motor neuron; LRP4, lipoprotein-related protein 4; M, muscle; MAA, myositis-associated Abs; MAG, anti-myelin-associated glycoprotein; MFS, Miller Fisher syndrome; MG, myasthenia gravis; miRs, myomiRs; miRNA, microRNA; MMNCB, multifocal motor neuropathy with conduction blocks; MRC, Medical Research Council; MRI, magnetic resonance imaging; MSA, myositis-specific Abs; MuSK, muscle-specific kinase; NcRNAs, Non-coding RNAs; Nf, neurofilament; NfH, neurofilament heavy chain; NfL, neurofilament light chain; NfM, neurofilament medium chain; NMD, neuromuscular diseases; NMJ, neuromuscular junction; NOS, nitric oxide synthase; NSAA, North Star Ambulatory Assessment; PM, polymyositis; PMP-22, peripheral myelin protein 22; pNfH, phosphorylated NfH; RYR, ryanodine receptor; SCLC, small cell lung cancer; SMA, spinal muscular atrophy; SMN, survival motor neuron; SOD1, superoxide dismutase 1; TDP-43, TAR DNA-binding protein 43; TMPRSS, transmembrane protease serine 5; TTNI, serum troponin; UMN, upper motor neuron; VGCC, voltage-gated calcium channel.
